# One year of hyperglycemia in the Ins2Akita mouse does not impart changes in retinal vascular patterning

**DOI:** 10.1371/journal.pone.0348363

**Published:** 2026-05-28

**Authors:** Fei Shang, Kosha Dholakia, Jesse Schallek

**Affiliations:** 1 Department of Neuroscience, University of Rochester, Rochester, New York, United States of America; 2 Center for Visual Science, University of Rochester, Rochester, New York, United States of America; 3 Department of Biomedical Engineering, University of Rochester, Rochester, New York, United States of America; 4 Flaum Eye Institute, University of Rochester, Rochester, New York, United States of America; Transilvania University of Brasov: Universitatea Transilvania din Brasov, ROMANIA

## Abstract

Diabetic retinopathy (DR) is one of the leading causes of blindness often associated with retinal vascular pathology in humans. To aid our understanding of DR, the Ins2^Akita^ mouse has served as a type 1 diabetes model. These mice have sustained elevated blood glucose (BG) and we tracked microvascular changes in the retinal circulation for up to one year. Retinal flat mounts of male Ins2^Akita^ x NG2DSRed (n = 14) mice age 7–61 weeks were imaged using a confocal microscope and compared to age matched euglycemic mice (n = 13) to establish a ground-truth of vascular patterning. We analyzed vessel density, vessel branching, vessel length, tortuosity, pericyte density, and the connectivity between vascular plexuses at eccentricities up to 2 mm from the optic disc. An additional hyperglycemic mouse was longitudinally imaged using an adaptive optics scanning light ophthalmoscope (AOSLO) for 12 weeks from 24–36 weeks of age. We found that up to one year of hyperglycemia had minimal impact on these retinal vasculature metrics that are also accessible to in vivo ophthalmoscopy. The longitudinally imaged mouse showed no changes and high congruence with ex vivo data. We conclude that yearlong elevated blood glucose did not impart detectable changes in retinal microvascular patterning.

## Introduction

Diabetic retinopathy (DR) is a common complication of diabetes mellitus (DM), affecting about 22.27% of the global diabetic population [1]. DR has a high societal and economic impact, with medical expenses alone estimated to be ~ $500 million in direct costs [2] with the worst outcomes leading to blindness in the working-age population [3]. Therefore, diagnosing DR as early as possible is vital to reducing this worst-case scenario.

Research has evolved from postmortem analysis of donor eyes [4] to in vivo fundus imaging and optical coherence tomography (OCT) of human patients [5–7]. From this work noted hallmarks of DR include: microaneurysms, dot and blot hemorrhages, hard exudates, cotton wool spots, leaky and occluded vessels, intraretinal hemorrhages, and vessel malformations such as venous beading and intra-retinal microvascular abnormalities (IRMAs) [8–10]. Some of the most recent work has deployed adaptive optics strategies to reveal pre-clinical tortuosity changes in the microvessels of diabetic patients without retinopathy [11].

However, DM patients undergo decades of vascular changes and are impacted by a myriad of factors including glycemic control, comorbidities such as hypertension and hyperlipidemia, and lifestyle factors including less physical activity [12–14]. This complicates our ability to isolate potential causal factors and their impact on the vasculature. Here, we begin with monitoring blood glucose (BG) and examining alterations in microvascular geometry.

Specifically, we used the Ins2^Akita^ mouse that models human type 1 diabetes. A missense mutation in the *Ins2* gene results in misfolded insulin protein and secondary β-islet dysfunction [15] due to toxic accumulation. These mice develop severe and sustained hyperglycemia (>400 mg/dL) from 4–5 weeks of age until death [16–18]. This is higher than in humans in which hyperglycemia can be classified at >125 mg/dL during fasting, > 180 mg/dL within 2 hours of eating [19], and >250 mg/dL can be considered very high [20]. These mice were then crossed with NG2DsRed mice which express a red fluorescent protein, DsRed, in cells with neuron-glial antigen 2 (NG2). Within the retina, this provides a transgenic vascular label that enabled clear visualization of the entire vascular tree and differentiation between arterioles, venules, and pericyte expressing microvessels [21,22]. All mice were on a C57BL/6J background as it’s the most commonly used strain of laboratory mice. We then deployed our optimized workflow for vascular metrics that can be utilized for both ex vivo and in vivo data [23].

The goal of this study was to assess if accessible vascular geometry metrics were altered in Ins2^Akita^ mice akin to human DR [24–26], and characterize any changes or lack thereof.

To address this goal, male Ins2^Akita^ (n = 14) mice aged 7–61 weeks were compared with age-matched euglycemic mice (n = 13). Ex vivo confocal microscopy provided the ground-truth basis for six metrics: 1) vascular density, 2) the number of vessel branches, 3) vessel length between bifurcations, 4) tortuosity, 5) pericyte density, and 6) the composition of axial vessels that span between lateral plexuses. These metrics were chosen as they can be non-invasively imaged with current ophthalmic techniques (e.g., red-free imaging [27], fluorescein angiography (FA) [28,29], optical coherence tomography-angiography (OCT-A) [28,29] and adaptive optics scanning light ophthalmoscope (AOSLO) imaging [30]). To show efficacy of in vivo study, an additional hyperglycemic mouse was examined for 12 weeks using AOSLO imaging.

We hypothesized that the sustained, elevated BG would correspond with some vascular changes. Thus, we analyzed the aforementioned six vascular metrics, across multiple eccentricities and up to one year of age. There were minimal detectable changes in these metrics and no microaneurysm formation observed. Contrary to our hypothesis, high BG did not correspond to microvascular change in the Ins2^Akita^ within the first year.

## Materials and methods

### Animals

This study used 29 Ins2^Akita^ X NG2DsRed mice (The Jackson Laboratory stock #:003548 and #008241, Bar Harbor, ME, USA), age 7–61 weeks. Twenty-seven of these mice were imaged exclusively ex vivo, 13 of which were euglycemic (BG < 200 mg/dL) and 14 hyperglycemic (>400 mg/dL). Two mice, a hyperglycemic and euglycemic pair, were imaged longitudinally from 24 weeks of age for 12 weeks. We exclusively used males due to the known female resistance to this hyperglycemic phenotype [16]. Blood glucose was measured weekly via a drop of blood from the tail vein and a OneTouch Ultra 2 meter (LifeScan Inc., Malvern, PA). Mice were housed under diurnal lighting conditions (12-hour light/dark cycle) and fed ad libitum. This study was carried out in accordance with the recommendations in the Guide for the Care and Use of Laboratory Animals of the National Institutes of Health. All guidelines from University Committee on Animal Resources at the University of Rochester, Rochester, New York were also followed.

### Ex vivo retinal imaging and analysis

Preparation, mounting, and imaging of the retinal tissue followed the same protocol as previously described [23]. In summary, mice were sacrificed and their retinas (n = 27) enucleated, fixed in 4% paraformaldehyde, flat mounted, and imaged using a confocal microscope (Nikon A1 Ti2 Eclipse). Montages of the entire retina were acquired at 20x magnification. For analysis, a minimum of four region of interest (ROI) Z-Stacks per mouse were captured at 60x magnification (300 μm x 300 μm) with a 0.1 μm axial step-size from four different eccentricities measured as millimeters from the optic disc (0 to 0.5, 0.5 to 1.0, 1.0 to 1.5, 1.5 to 2.0 mm).

Z-Stacks were traced using ImageJ Neuroanatomy SNT plugin. While tracing and during analysis, a vessel segment was defined as the length between nodes, i.e., branch points or bifurcations. Vessels were traced such that each vessel segment was its own individual path within SNT.

X, Y, and Z trace coordinates were then exported and analyzed using custom MATLAB code available on GitHub [31]. Quantified metrics included: vessel density (mm/mm2), vessel segments per area (count/mm2), vessel length (μm), tortuosity (vessel length/shortest path), pericyte density (count/mm2), and axial vessel composition (SI connecting, ID connecting, SID connecting, SD connecting, inter-plexus branching).

### In vivo retinal imaging and analysis

Mice were anesthetized and imaged using both commercial scanning light ophthalmoscope (SLO) and adaptive optics scanning light ophthalmoscope (AOSLO) imaging. Anesthesia was induced via a ketamine-xylazine mixture (intraperitoneal injection (IP): 100 mg/kg ketamine, 10 mg/kg xylazine) and sustained with 1% v/v isoflurane delivered continuously via a nose cone. Prior to all imaging sessions, sodium fluorescein was injected (IP: 0.1 mL; 10:1 dilution of 100 mg/ml 10% AK-FLUOR; Akorn, Lake Forest, IL) and pupils were dilated with eye drops of 1% tropicamide (Sandoz, Basel, Switzerland) and 2.5% phenylephrine (Akorn, Lake Forest, IL). A + 20 D rigid contact lens (1.6 mm base curvature, Advanced Vision Technologies, Lakewood, CO) covered the cornea and GenTeal (Alcon Laboratories, Inc., Fort Worth, TX) maintained hydration. Mouse body temperature was kept at 37° C via an electric heating pad.

For orientation and qualitative assessment of global changes, SLO imaging was captured two days prior to the first and last AOSLO sessions. Using AOSLO imaging, videos (10 s) of the nerve fiber layer recorded from the optic disc to ROIs created higher resolution, localized maps. These maps use the optic disc and large vessels as landmarks to return to the same locations over time. During acquisition, cartesian videos were captured within 0.5 mm of the optic disc and between 0.5 mm and 1.0 mm from the optic disc, relative to first order blood vessels. At each ROI, 13–14 videos (30 s) were captured at discrete axial steps by changing the defocus on the deformable mirror. This translated to approximately 7.31 μm based on the average thickness of this region of 95 μm [32]. These ROIs were tracked over 3 imaging sessions spanning 12 weeks.

A custom MATLAB script registered videos by using normalized cross-correlation to compare each frame to a manually selected ROI [33] for each individual video. The videos were then averaged into single images, contrast stretched manually and combined into a Z-Stack. During this post processing, data quality was assessed and Z-Stacks that did not show multiple vessel beds due to poor signal-to-noise were not included in the analysis. Z-Stacks of sufficient quality were traced identically to ex vivo histology. After the final imaging session, the retina was fixed, flat mounted and imaged at the same ROIs ex vivo (n = 1).

#### Widefield fundus imaging.

We used the SPECTRALIS (Heidelberg Engineering Inc., Heidelberg, Germany) for SLO imaging centered at the optic disc (55° field of view (FOV)). It used 815 nm infrared reflectance (IR mode) and 488 nm excitation (fluorescein angiography (FA mode)). In FA mode, fundus imaging was performed to capture the superficial, intermediate and deep capillary plexuses with a 20-frame automatic real time averaging (ART).

#### AOSLO imaging.

A custom built AOSLO designed for the mouse eye [18,34] was used to image simultaneous phase contrast and fluorescence channels to capture cartesian videos (FOV: 5.01° × 3.89°) at multiple ROIs. In brief, this instrument uses three axially combined light sources: 1) Near infrared reflectance (NIR): 796 nm superluminescent diode (214 µW, Superlum, Cork, Ireland); 2) Fluorescence: 488 nm (56 µW, Toptica Photonics, Farmington) and 3) wavefront sensing: 904 nm laser diode (12 µW, Qphotonics, Ann Harbor, MI) were axially combined and relayed to the mouse eye through five afocal telescope pairs in a raster scan pattern generated with a 15 kHz resonant scanner and 25 Hz galvanometer scanner. The mouse eye’s optical aberrations were detected using a Shack Hartman Wavefront Sensor then corrected with a 97-actuator deformable mirror (ALPAO, Montbonnot-Saint-Martin, France). Light from the mouse retina was captured with two photomultiplier tubes (Hamamatsu Photonics, Hamamatsu, Japan) set in 2 different configurations: 1) near infrared light was detected with a H7422-50 PMT with an offset aperture [35] and 2) simultaneous confocal fluorescence detection with a H7422-40 PMT through a 520Δ35 band-pass filter (FF01-520/35-25, Semrock, Rochester, NY) and 25 μm pinhole.

### Statistical analysis

All statistics were calculated using GraphPad Prism version 10.4.2 for Windows (GraphPad Software, Boston, Massachusetts, USA). All results were considered significant at P < 0.05. Values were reported as the mean ± 1 standard deviation.

Weekly blood glucose and weight measurements were collected from each mouse. Due to the nature of progressive sacrifice of the population of mice over time, Prism’s mixed-effects analysis variation of repeated measures ANOVA was used with a Geisser-Greenhouse correction as we did not assume sphericity.

During initial acquisition some retinas were imaged more than others. To prevent individual retinas from having greater contribution to the data without arbitrarily excluding images, multiple Z-Stacks from the same retina in the same eccentricity range were averaged into one value. The first comparisons were made with all Z-Stacks averaged into one value per animal. For each vascular metric, unpaired two-tailed Welch’s t tests were used for comparisons. It compared the summed totals across all vascular layers for each metric between euglycemic and hyperglycemic mice. This provided a holistic analysis of all the data. Welch’s t-test was used to increase power in the case of unequal variance between the groups, at the risk of decreasing power if the variance was equal. Due to the relatively high variability [23], this was considered the less likely possibility. Two-way ANOVAs for each metric were then run to examine blood glucose and trilaminar layer effects and interactions. Each mouse again represented by the average value from all its Z-Stacks. After all ANOVAs, post-hoc Tukey’s was used.

For age comparisons, Z-Stacks from the same retina were averaged, binned by age (<20 weeks, 20–30 weeks, 30–40 weeks, 40–50 weeks, > 50 weeks), and analyzed with two-way ANOVAs. For eccentricity comparisons, 4 Z-Stacks per retina were binned by eccentricity (0 to 0.5 mm, 0.5 to 1.0 mm, 1.0 to 1.5 mm, 1.5 to 2.0 mm) and analyzed with mixed-effects ANOVAs to account for matching. Three-way ANOVAs assessed the combined impact of age and eccentricity with blood glucose. They were run with simplified age brackets of young adult (<30 weeks) and middle-aged (>30 weeks) due to prism restrictions for three-way ANOVAs. This also increased the sample size of the age comparison.

Correlation analysis using Pearson’s R and simple linear regression were used to determine the relationship between BG severity and vascular metrics. Runs test confirmed the assumption of linearity, so we did not run any non-linear regression analyses.

## Results

### Ins2^Akita^ mice exhibit sustained hyperglycemia

**Fig 1:**
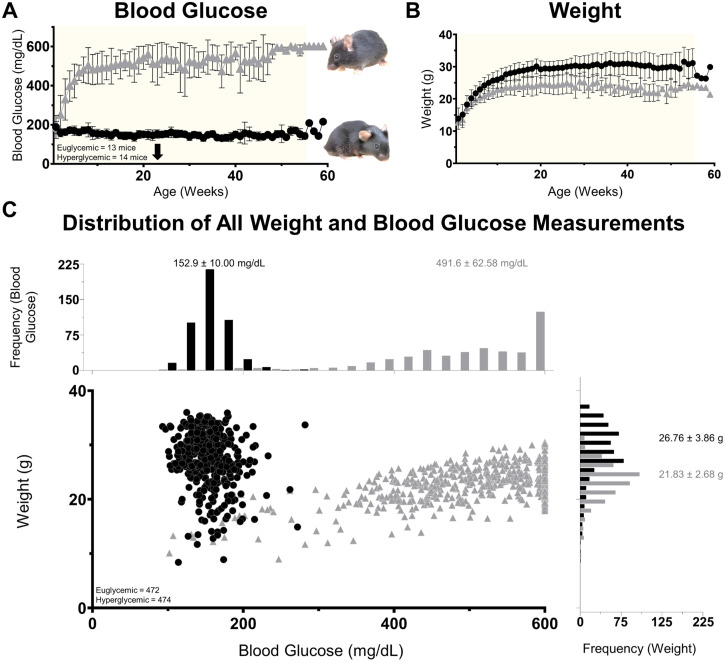
Two distinct mice populations separated by blood glucose. **(A)** Blood glucose as a function of age shows an early and large difference. At right: example images of each mouse (22 weeks, black arrow). S1 Video shows their similar activity level. **(B)** Weight as a function of age shows hyperglycemic mice have minor weight loss. The light background highlights data with multiple mice as the last four points represented only the oldest pair. **(C)** Plot of 946 BG and weight measurements taken from 13 euglycemic and 14 hyperglycemic mice. Histograms along each axis show high overlap in weight and low overlap in BG level with the average and standard deviation reported for each.

Onset of hyperglycemia occurred at postnatal week 4 (Fig 1A; blood glucose main effect F(1,25)=723.5; post-hoc Tukey’s P = 0.009) and remained elevated. Average BG levels were 152.9 ± 10.00 mg/dL for euglycemic (E) mice and of 491.6 ± 62.58 mg/dL for hyperglycemic (H) mice. These values matched with prior reports from healthy C57BL/6J mice [36,37] age 24–72 weeks [38] and Ins2^Akita^ mice [15–18] respectively. However, the severity of hyperglycemia might be underestimated in our mice, especially at older ages, as BG levels often reached the ceiling of our glucose meter (600 mg/dL). Furthermore, HbA1c was not measured as its value is positively correlated with BG and associates with the average BG level of a two-week time period [39]. Thus, we consider the direct BG measure more informative with finer temporal resolution.

Mice were also weighed at the time of each BG measurement. Unlike type 2 diabetic models like db/db mice [40] and diet induced models [41], Ins2^Akita^ mice exhibited mild weight loss by week 7 (Fig 1B; P = 0.015). This also aligned with previous Ins2^Akita^ reports [15–18]. Although there were signs of polydipsia and polyuria in the bedding of their cages, the outward appearance and behavior of adult hyperglycemic mice was hard to distinguish from littermate controls (S1 Video) suggesting a hyperglycemic phenotype with limited systemic complications.

### No overt ex vivo differences between euglycemic and hyperglycemic retinas

**Fig 2:**
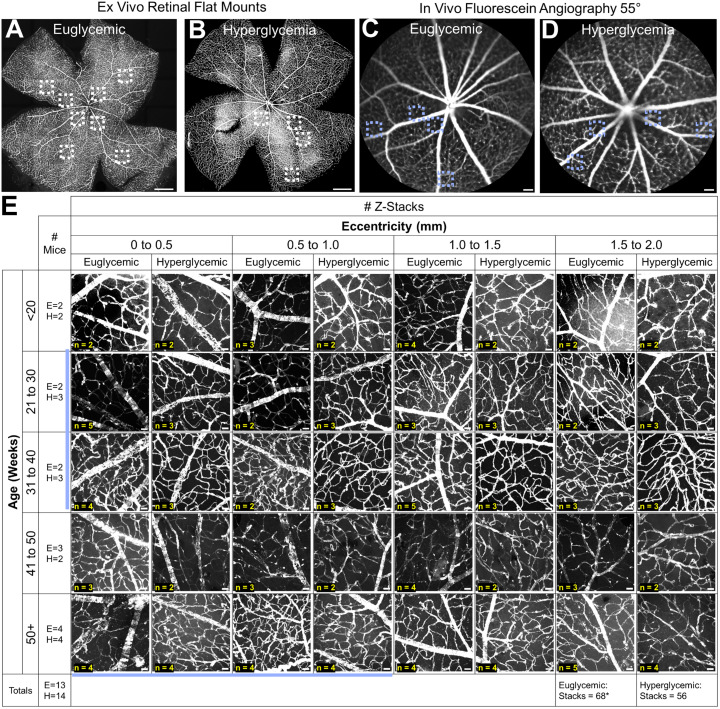
Imaging paradigm and examples from euglycemic and hyperglycemic mice. Example ROI (outlined by dotted boxes) distributions from four different mice. **(A-B)** 20x magnification montages. Scale bar = 500 μm. **(C-D)** 55° FOV fluorescein angiography. Scale bar = 100 μm. **(E)** Representative ex vivo Z-Stacks for both glycemia groups organized by eccentricity (horizontal) and age (vertical). The number of male mice for each age range is in the left column, while the number of Z-Stacks for each age and eccentricity group is within each box. Scale bar = 25 μm. Light blue lines highlight the analysis bins corresponding to the in vivo hyperglycemic mouse data. Contrast adjustment for visualization purposes, original images available upon request. *One euglycemic mouse was analyzed using a montage from optic disc to ora serrata; it was included in the total but not in any of the eccentricity by age boxes.

The ex vivo data set included 27 flat mounted retinas from male mice age 7–61 weeks (E: n = 13; H: n = 14) with detailed analysis from 124 high-resolution Z-Stacks. Example images and analysis bins utilized during quantification is summarized in Fig 2.

Similar to healthy mice [22], the NG2DsRed labeling was bright and sustained for over a year in both populations. At 60x magnification, the differing NG2 patterning that distinguishes venules, arterioles, and capillaries from one another [22] was preserved. There was also no abrupt cessation of labeling and all three vascular layers and their interconnecting vessels were visible. This indicated that hyperglycemia did not impact NG2 protein expression nor our ability to detect change in the quantitative metrics below. We also saw no visible retinal pathology. This included no apparent vessel dropout, remodeling in any of the layers or in any specific location, microaneurysms, IRMAs or collapsed vessels. This suggested intact perfusion which is supported by our previous work that showed functional capillaries in the Ins2^Akita^ mouse [18]. The full dataset is displayed in S1 and S2 Fig.

### Limited impact of hyperglycemia on six quantified vascular metrics

Since it is possible that gain, loss, and repatterning of vessels is dynamic throughout the course of hyperglycemia, we quantified six key vascular metrics that are often imaged in vivo at different time points. First, we examined each metric holistically by collapsing all retinal vessels into a two-dimensional sheet similar to what would be viewed with en face fundoscopy. Second, layer specific changes were evaluated, comparable to emerging capabilities in AOSLO and OCT-A. In mouse, like many regions in human retina, these layers exist as three plexuses of superficial (S), intermediate (I), and deep (D) vessels. These localize approximately to the nerve fiber layer (NFL), inner plexiform layer (IPL), and outer plexiform layer (OPL) respectively. Interconnecting them are few but important axial vessels that lie between the superficial and intermediate (SI) and the intermediate and deep (ID) layers. Third, we considered the interactions of eccentricity and age. Finally, the severity of BG level was plotted against each vascular metric for each mouse. Across all these analyses, minimal change was observed.

#### Vessel density, branching, length, and tortuosity.

**Fig 3:**
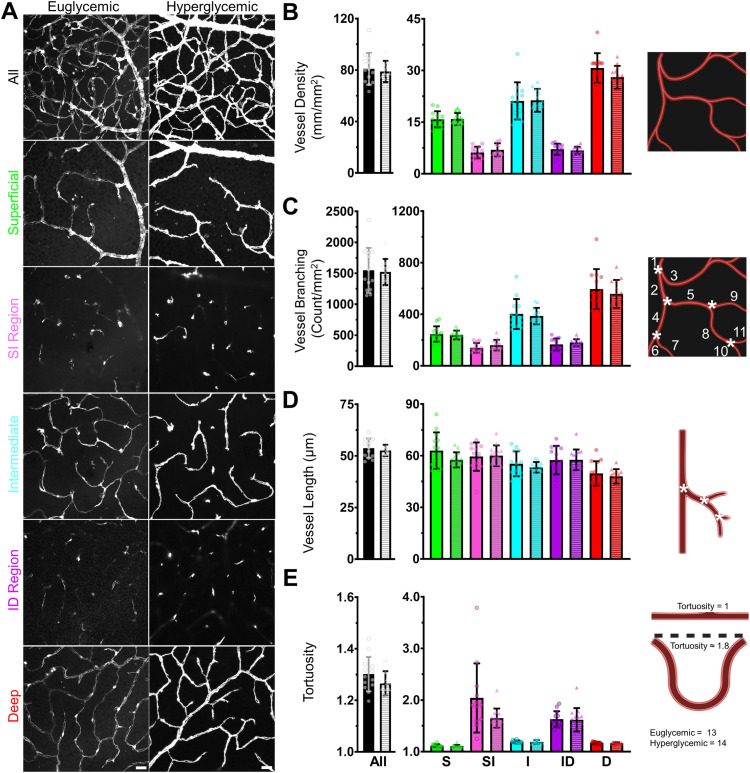
Vascular metrics were not significantly different between groups. **(A)** Age and eccentricity matched examples from each glycemia group. The first row is a projection of the entire Z-Stack. Each row beneath is a projection of a subset of the Z-Stack corresponding to the superficial, SI, intermediate, ID, and deep layers. Scale bar = 25 μm **(B)** Vessel density, **(C)** vessel branching, (D) vessel length, and (E) tortuosity quantification. (B-E) are divided into layer total (left) layer breakdown (center), and a visual diagram created with Biorender.com (right). White asterisks denote nodes between vessel branches or segments. One extreme outlier in the euglycemic data was omitted for visualization purposes. We used Welch’s unpaired t-test for total and two-way ANOVAs with post-hoc Tukey’s for each vascular layer.

We did not find a significant difference for metrics which characterize overall vascular patterning. Vessel density (E: 80.92 ± 12.44 mm/mm^2^; H: 78.91 ± 8.26 mm/mm^2^; Welch’s unequal variance t-test P = 0.629; Fig 3B), vessel branching (E: 1548 ± 362.8 count/mm^2^; H: 1522 ± 210.8 count/mm^2^; P = 0.828; Fig 3C), vessel length (E: 53.75 ± 4.63 μm; H: 52.64 ± 2.75 μm; P = 0.464; Fig 3D), and tortuosity (E: 1.30 ± 0.07; H: 1.27 ± 0.05; P = 0.126; Fig 3E) were similar in both euglycemic and hyperglycemic mice. This suggests that traditional in vivo imaging would also not detect changes and matches with our initial qualitative observations.

The layer-by-layer analysis also showed no visible differences between glycemia groups. Both populations showed similar overall patterning with greater vessel density and branching in the deeper plexuses. Additionally, vessel length was similar between all trilaminar layers. This supported our previous conclusion [23], that there is a degree of consistency in vessel length throughout the retina, and that consistency was not measurably altered by hyperglycemia. When quantified using two-way ANOVAs with glycemia group and trilaminar layer as the two cofactors and post-hoc Tukey’s, no significant differences were revealed (Table 1; Fig 3).

**Table 1:** Summary of vascular metrics for euglycemic and hyperglycemic mice.

		Total	S	SI	I	ID	D
Vessel Density (mm/mm^2^)	E	80.92 ± 12.44	15.81 ± 2.35	6.14 ± 1.72	21.16 ± 5.41	7.09 ± 1.64	30.73 ± 4.31
	H	78.91 ± 8.26	15.88 ± 1.76	6.93 ± 1.91	21.33 ± 3.34	6.74 ± 1.10	28.04 ± 3.31
	P	0.6286	0.9263	0.2697	0.9234	0.5180	0.0840
Vessel Branching (Count/mm^2^)	E	1548.00 ± 362.80	246.20 ± 59.74	139.10 ± 37.58	401.90 ± 116.50	165.40 ± 46.04	595.00 ± 155.10
	H	1522.00 ± 210.8	238.50 ± 34.49	160.20 ± 41.08	385.80 ± 63.83	180.00 ± 26.50	557.70 ± 108.70
	P	0.8276	0.6916	0.1766	0.6639	0.3286	0.4793
Vessel Length (μm)	E	53.75 ± 4.63	62.98 ± 10.57	59.48 ± 8.23	55.28 ± 7.27	57.44 ± 8.31	49.71 ± 7.05
	H	52.64 ± 2.75	57.61 ± 4.36	59.95 ± 6.03	53.24 ± 3.07	57.60 ± 6.03	48.02 ± 4.16
	P	0.4639	0.1084	0.8672	0.3615	0.9547	0.4630
Tortuosity	E	1.30 ± 0.07	1.11 ± 0.03	2.63 ± 2.21§2.04 ± 0.67	1.19 ± 0.02	1.63 ± 0.15	1.17 ± 0.02
	H	1.27 ± 0.05	1.11 ± 0.02	1.65 ± 0.19	1.19 ± 0.04	1.62 ± 0.23	1.16 ± 0.02
	P	0.1259	0.3757	0.1378 0.0733^a^	0.5979	0.8791	0.5121
Pericytes (Count/mm^2^)	E	634.90 ± 116.4	83.50 ± 21.22	69.92 ± 26.94	207.20 ± 45.33	59.62 ± 18.55	214.60 ± 51.73
	H	654.10 ± 103.6	102.30 ± 22.61	81.81 ± 18.54	206.70 ± 40.91	56.50 ± 13.13	206.70 ± 42.39
	P	0.6571	*0.0355	0.1991	0.9763	0.6206	0.6698
Pericyte Ratio (μm/pericyte)	E	129.50 ± 14.89	230.10 ± 57.98	130.60 ± 39.57	105.60 ± 15.23	206.40 ± 82.00	140.70 ± 40.43
	H	122.40 ± 12.89	174.10 ± 73.93	127.00 ± 29.24	98.79 ± 13.15	220.70 ± 51.88	130.40 ± 17.20
	P	0.1980	*0.0376	0.7940	0.2290	0.5977	0.4105

E rows are euglycemic mice (n = 13) averages ± SD, H rows are hyperglycemic mice (n = 14) averages ± SD, P rows are p-values. P-values in Total column are calculated from unpaired two-tailed Welch’s t-test while individual trilaminar layers were calculated from two-way ANOVAs post-hoc Tukey’s analysis. *P < 0.05.

a Recalculated with a single extreme outlier excluded

We also did not see elevated BG differentially impact any of these metrics based on vascular layer except for tortuosity (F(4,125)=2.635; P = 0.037). However, the magnitude of the mean differences between the tortuosity of different layers was similar for both groups, with the greatest disparity being less than 1 (S1 Appendix). Thus, the current data does not support this being biological meaningful despite statistical significance.

#### Pericytes.

The next metric examined was pericytes as prior reports emphasize pericyte loss as a key factor in DR [4] and emerging approaches seek to quantify pericytes in vivo [42]. As NG2 is not exclusive to pericytes, we paired the fluorescent signal with morphology, i.e., the protrusion of a soma with extending processes. We then reported our findings in terms of density (count/mm^2^) and coverage as a ratio of vessel length between pericytes (μm/pericyte).

**Fig 4:**
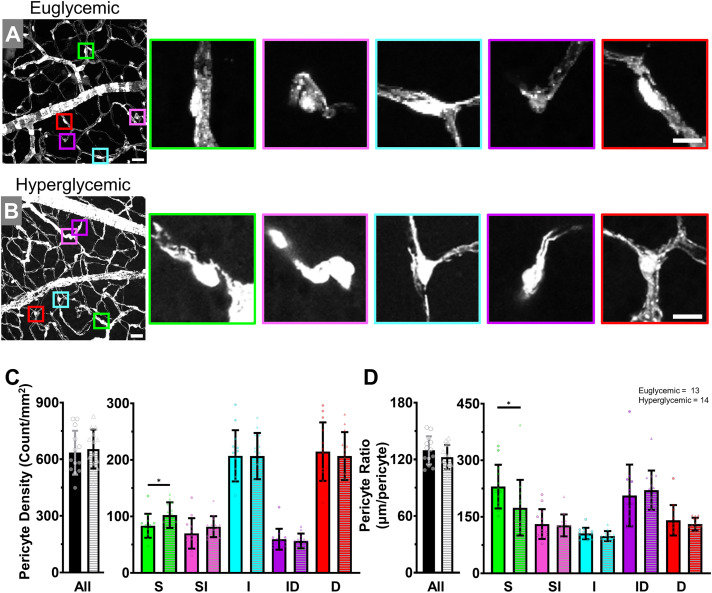
Pericyte density is similar between euglycemic and hyperglycemic mice. **(A)** Euglycemic or (B) hyperglycemic Z-Stack with colored squares highlighting locations of pericyte examples in each of the vascular layers. Scale bar = 25 μm. Enlarged images of the pericytes are arranged in increasing depth from left to right. Scale bar = 10 μm. **(C)** Pericyte density and (D) vessel length to pericyte soma ratio quantification. Welch’s unpaired t-test for all and two-way ANOVAs with post-hoc Tukey’s for each vascular layer. *P < 0.05.

We found the overall euglycemic pericyte density (Fig 4A, 4B) was 634.9 ± 116.4 pericytes/mm^2^, matching our prior report [22]^.^. Neither the total hyperglycemic pericyte density (H: 654.1 ± 103.6 pericytes/mm^2^; P = 0.657; Fig 4C) nor the ratio of pericyte coverage was significantly different (E: 129.5 ± 14.89 μm/pericyte; H: 122.4 ± 12.89 μm/pericyte; P = 0.198; Fig 4D).

When examined across layers, we did not find significant differences in capillary pericyte density between groups. In terms of patterning, pericytes were fewest in number in the superficial plane with greater numbers in the intermediate and deep layers. This organization matched the greater vessel density described above. As such, this balance was captured in the pericyte coverage ratio too (Fig 4D), with greater length of vessel between pericytes in the superficial layer compared to the intermediate and deep layers.

Only the superficial layer had a significant difference between groups for both pericyte density (E: 83.50 ± 21.22; H: 102.30 ± 22.61; P = 0.036; Fig 4C) and coverage (E: 230.10 ± 57.98; H: 174.10 ± 73.93; P = 0.038; Fig 4D). This difference was likely due to the presence of larger vessels within the superficial layer. However, like the above analyses, there was no significant covariation of glycemic state and trilaminar layer. Thus overall, both the vascular patterning and mural cells were not detectably altered by sustained hyperglycemia.

#### Microvessel connections between plexuses.

**Fig 5:**
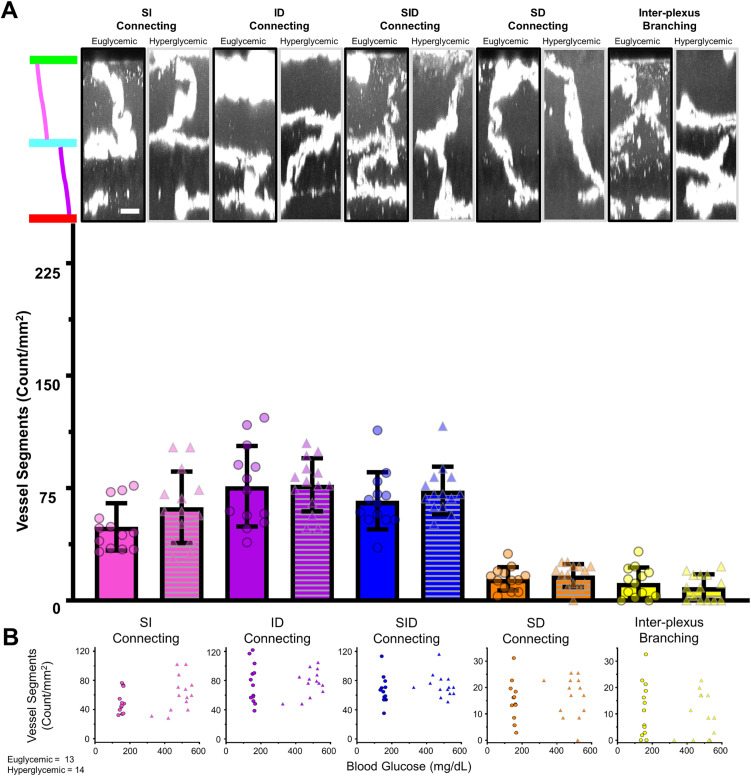
Interconnecting microvessels did not show significant changes. **(A)** Euglycemic and hyperglycemic examples of each of the 5 categories of axial vessels. Images are aligned with a colored diagram on the left showing the vascular layers. Scale bar = 25 μm Beneath is the corresponding quantification of the number of axial vessels per area. Two-way ANOVAs with post-hoc Tukey’s used for each axial vessel type. **(B)** Plots of the relationship between BG severity and number of axial vessels for each category.

The sixth metric examined the vessels often missed with both in vivo imaging, due to their mostly perpendicular orientation to the imaging plane, and trypsin digest preparations, due to axial collapse. Their value is immense as they are the sole connections relaying blood between the three plexuses, and early microscopic changes may profoundly impact perfusion. Figure 5 shows the different types of connective vessels that bridge the vascular plexuses. SI connects the superficial and intermediate, ID connects the intermediate and deep, SID connects all three layers, SD forms a direct bridge from superficial to deep, and inter-plexus branching bifurcates within one of the nuclear layers.

We did not find a significant difference in any of the 5 types between euglycemic and hyperglycemic mice as summarized in Table 2. Similarly, we did not find a rebalancing or redistribution of the types, suggesting a lack of remodeling. This was further supported by a lack of covariance between glycemic state and axial vessel type.

**Table 2:** Densities of interconnecting microvessels.

(Count/mm^2^)	Euglycemic	Hyperglycemic	P-value
SI Connecting	49.12 ± 15.77	62.17 ± 23.81	0.1048
ID Connecting	76.16 ± 26.88	77.15 ± 17.73	0.9120
SID Connecting	66.46 ± 19.01	73.30 ± 15.95	0.3231
SD Connecting	14.42 ± 7.77	16.60 ± 7.61	0.4673
Inter-PlexusBranching	11.72 ± 10.22	8.91 ± 8.53	0.4470

P-value calculated from two-way ANOVA post-hoc Tukey’s analysis.

#### No eccentricity dependent changes detected in response to hyperglycemia.

In humans, DR changes often initiate at specific locations and expand over time [8–10]. Thus, we examined the above metrics in an eccentricity dependent fashion to test whether specific distances from the mouse optic disc were differentially impacted. We used two-way ANOVAs to assess any covariance between glycemic state (E: n = 12; H: n = 14) and eccentricity and their impact on each of the six metrics. Overall, we found similar density, branching, length and tortuosity (S3 Fig); pericyte density (S5 Fig A); and axial vessel composition (S6 Fig A) between the two groups regardless of distance from the optic disc.

There was a lack of strong, consistent changes that would suggest an eccentricity-dependent effect which is aligned with what we observed by eye. However, there were some subtle effects such as significant covariance between glycemic state and eccentricity in the following metrics: branching (F(3,72)=3.831; P = 0.013) and length (F(3,72)=2.965; P = 0.038) in the ID region, overall pericyte ratio (F(3,72)=3.337; P = 0.024), and number of SID connections (F(3,72)=3.28; P = 0.026). Post-hoc Tukey’s revealed both increased (SI vessel density: P = 0.031; SI Length: P = 0.047; superficial pericytes density: P = 0.024) and decreased (deep vessel density: P = 0.0495; superficial tortuosity: P = 0.039; total pericyte ratio: P = 0.017; superficial pericyte ratio: P = 0.033; intermediate pericyte ratio: P = 0.004) metrics in the hyperglycemic mice spread across a range of eccentricities (0.5–1.0 mm, 1.0–1.5 mm, and 1.5–2.0 mm). Thus, while we observed no specific bias of changes in either the central or peripheral retina, minute alterations may be occurring on an individual basis in isolated areas.

#### By one year, limited vascular changes were detected.

To look for overall changes in vascular patterning, we averaged each metric across all eccentricities. In the oldest mice (50 + weeks), there were no significant differences between euglycemic (n = 3) and hyperglycemic (n = 4) mice in any metric for any vascular layer. This suggested that despite prolonged hyperglycemia there was no measurable change in the geometry of the retinal vasculature. However, we also wanted to examine potential subtle differences that might have occurred throughout the time-course of these mice.

To do this, we ran three-way ANOVAs to examine the interaction of age, eccentricity and glycemic state. The ANOVAs were conducted on pooled data from two age epochs, younger adult mice (≤30 weeks; E: n = 5; H: n = 6) and “middle-aged” mice (>30 weeks; E: n = 7; H: n = 8). As a single variable, glycemic state was not a significant source of variation for any metric. There was also no significant covariance involving all three factors. There were a few instances in which age and glycemic state were significant together (total vessel density (F(1,22)=6.161; P = 0.021), SI vessel density (F(1,22)=7.818; P = 0.011), deep vessel density (F(1,22)=4.534; P = 0.045), total number of branches (F(1,22)=5.505; P = 0.028), deep number of branches (F(1,22)=6.030; P = 0.022), deep vessel length (F(1,22)=6.599; P = 0.018), intermediate tortuosity (F(1,22)=7.660; P = 0.011), and number of IPB axial vessels (F(1,22)=5.713; P = 0.026). This suggested that hyperglycemia may have impacted the vasculature differently depending on the age examined, but our current data did not show any clear patterns. All t-test, ANOVA, and post-hoc results are reported in S1 Appendix**.**

#### Severity of BG does not associate with vascular metrics.

**Fig 6:**
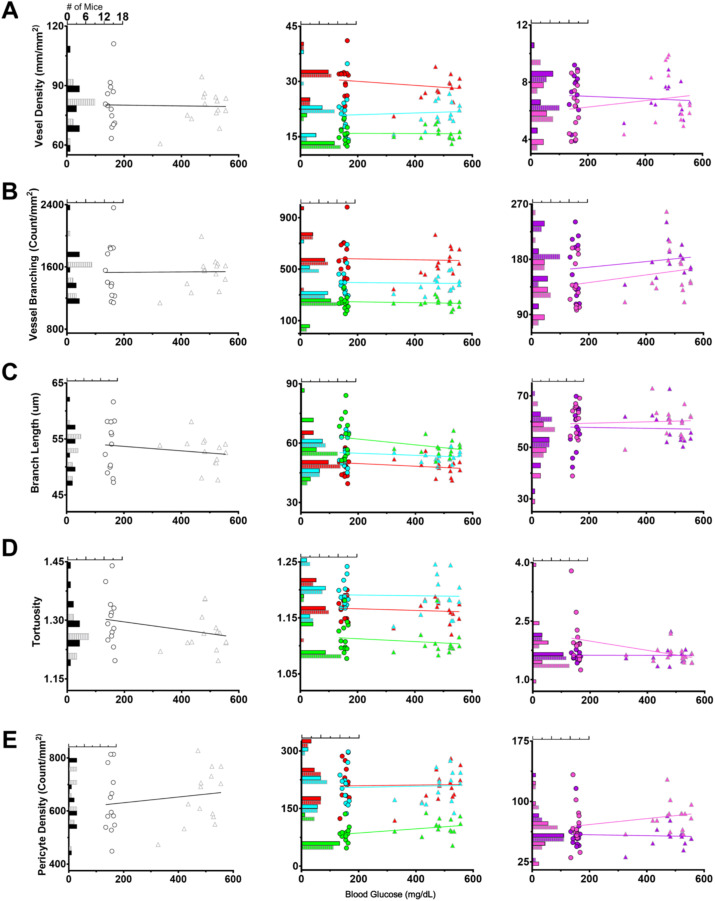
Blood glucose severity is a poor predictor of vascular changes. (A) vessel density, (B) vessel branching, (C) vessel length (D) tortuosity, and (E) pericyte density correlations between the average value and average BG. Shown with linear regression lines. The columns from left to right: total; superficial, intermediate, and deep; SI and ID quantifications. Embedded in the y-axis are histograms of the number of mice to show the distribution of average values.

The previous analyses examined differences in vascular patterning based on two mouse populations. However, it is possible that BG may be directly associated with these vascular metrics. Therefore, we examined the correlation between BG severity and each of the vascular metrics for each mouse. We assessed BG severity in two ways: 1) the average BG level of each mouse across its lifespan and 2) the number of weeks BG level was > 400 mg/dL. The second measure was an attempt to disambiguate age from duration of hyperglycemia. Despite up to a threefold difference in BG level creating two distinct clusters (Fig 1 and Fig 6), we found little correlation with any analyzed metric.

There were a few significant correlations. These included two for average BG level (superficial pericyte density (R = 0.41; P = 0.036) and the number of SI connecting vessels (R = 0.4; P = 0.041)) and two for duration of hyperglycemia (SI vessel density (R = 0.43; P = 0.026) and number of SI connecting vessels (R = 0.39; P = 0.046)). Of note, pericyte density in the superficial layer has high regional variability due to the presence or absence of large blood vessels in the region analyzed, so we consider this first correlation with caution. The other three correlations suggest the SI region could be more vulnerable to BG-mediated changes, but larger cohort studies will be needed.

Our second approach to examining the impact of BG severity was to isolate this analysis to only the hyperglycemic population. Now, we saw four for BG level (intermediate vessel density (R = 0.59; P = 0.027), deep vessel density (R = 0.62; P = 0.018), number of deep branches (R = 0.61; P = 0.019), and deep pericyte density (R = 0.68; P = 0.007) and four for duration of hyperglycemia (SI vessel density (R = 0.59; P = 0.026), ID vessel density (R = 0.71; P = 0.005), total vessel density (R = 0.59; P = 0.025), and intermediate tortuosity (R = 0.55; P = 0.0). The full list of correlations can be found in S2 Appendix. These additional correlations may indicate other more vulnerable vascular layers such as the intermediate and deep plexuses.

However, variation in the average value of each vascular metric had a similar distribution despite the great disparity in BG level (Fig 1 and Fig 6). This variability paired with the weak correlations, suggested that sustained elevated BG level had a limited impact.

### In vivo imaging showed no change in vascular structure in a hyperglycemic mouse

**Fig 7:**
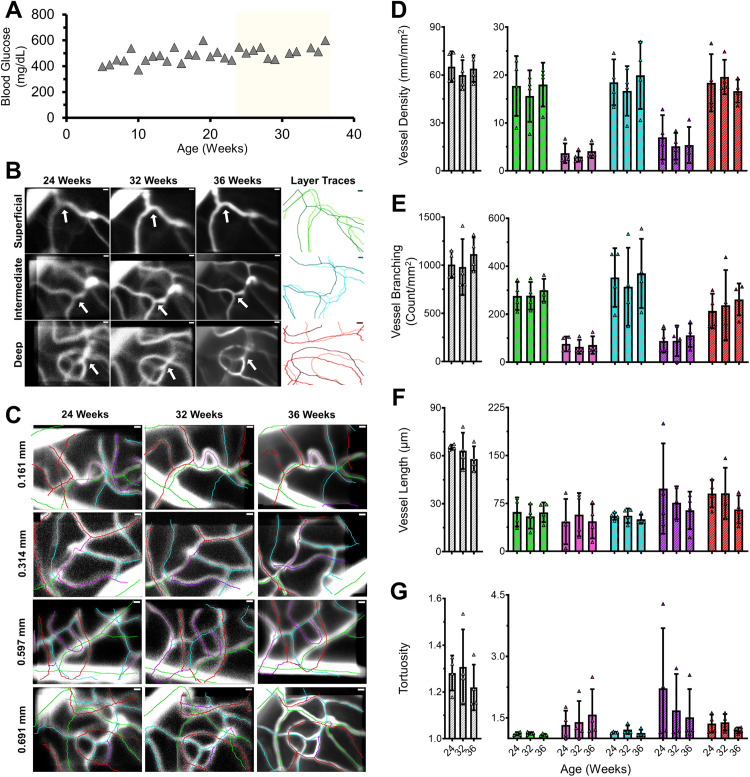
Vessel structure was similar across 12 weeks of elevated blood glucose. **(A)** Weekly BG measurements, yellow shading is placed over the time window imaged. **(B)** Projections of in vivo Z-Stacks from each vascular layer of one ROI at each time point. Far right column shows the SNT traces to highlight the matching features and variability between imaging sessions. The trace color darkens for each progressive time point and all three are aligned by their top right corner. White arrows highlight shared landmarks between sessions. **(C)** All hyperglycemic in vivo data showing all layers, locations, and time points. SNT traces are overlayed. Scale bar = 10 μm. Images were contrast adjusted for visualization purposes; original images available upon request. Graphs of 4 vascular metrics: (D) vessel density, (E) vessel branching, (F) vessel length, and (G) tortuosity. Each dot represents one of four locations within the same mouse.

To track vascular patterning over time, akin to repeated visits in a clinical setting, we used adaptive optics scanning light ophthalmoscopy (AOSLO) to image one mouse for 12 weeks from 24–36 weeks of age**.** At all locations imaged, we could return to the same location and found there were no changes visible in the sodium fluorescein signal (Fig 7).

Not only did we observe the same vascular pattern at all four locations over time, but the quantified metrics averaged only a 5.68% difference between timepoints. As there were no overt structural changes or changes in perfused vessels, this difference was more likely due to inescapable variance between imaging sessions rather than a biologically meaningful difference. This was further supported by the similarity to previous in vivo euglycemic data [23] and one healthy littermate (S7 Fig).

**Fig 8:**
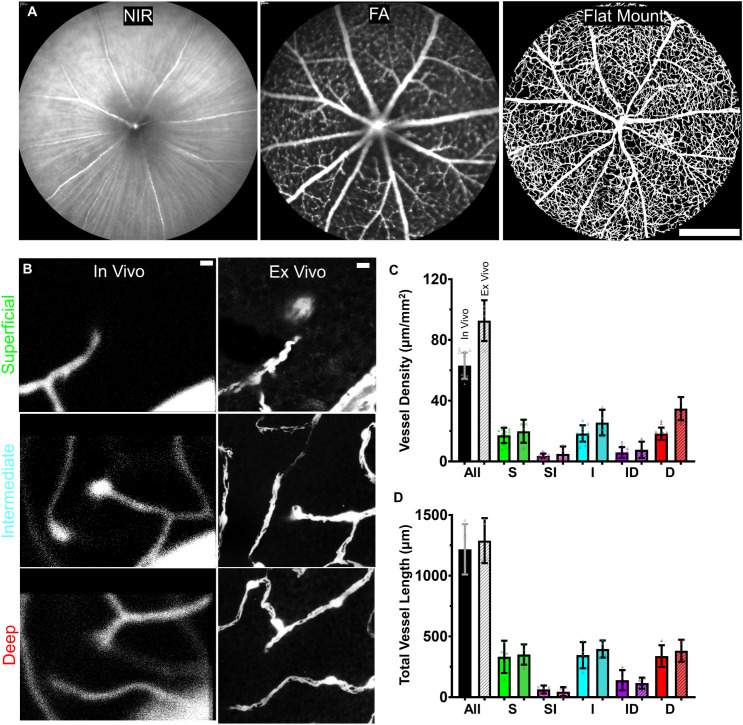
In vivo and ex vivo data showed good agreement. **(A)** NIR, FA, and flat mount images of the same retina centered on the optic disc. Scale bar = 500 μm **(B)** Images from the three trilaminar layers at the same location in vivo (left) and ex vivo (right). Scale bar = 10 μm **(C)** Vessel density quantification where each location and time was represented in vivo (n = 12) and compared to each location ex vivo (n = 4). **(D)** Vessel length traces for one-to-one vessel length comparison. Due to FOV movement, values were averaged across all time points for in vivo locations (n = 4) and compared to the same location ex vivo (n = 4).

An added advantage of our experimental design meant we could also examine the same anatomical location after death (week 37). This allowed both longitudinal study and ex vivo validation (Fig 8). We found that the complete 3D visualization of the same locations matched exceptionally well. Vessel lengths only differed by 6.29%, which we considered small given the remarkably different preparations. As the overall patterning was visually similar, we suspected this mismatch was likely due to either histological artifact or unforeseen small scaling factors that differed between the approaches.

## Discussion

Despite exceptionally high BG levels established by week 4 in the Ins2^Akita^ mouse, we found few changes in the patterning of retinal vessels. Our report focused on six foundational metrics that are becoming increasingly available to clinical study through the advance of high-resolution imaging. This result contrasts with the vast literature that supports the finding in humans that prolonged exposure to high BG imparts changes in the microvasculature of the retina [4,8–11,42,43]. Here, we discuss several considerations and contexts to understand these results.

Compared to other work using Ins2^Akita^ mice, our mouse colony had similarly rapid and severe hyperglycemia starting at week 4, highlighting the robustness of this phenotype. Barber et al. [17] was among the first to examine retinal complications in this model. Like our study, they also examined pericytes but focused on pericyte ghosts [17] seen with trypsin digest. While we did not perform trypsin digest, our assay instead preserved the natural 3D architecture of the retina. Using the NG2 DsRed marker, we found the same number of pericytes in both euglycemic and hyperglycemic mice, which matched a prior report from our lab [22]. As this fluorescent protein is only produced with a functioning nucleus and viable protein transcription, we interpreted an NG2 + pericyte as a living mural cell. There is some danger in this interpretation as DsRed has a relatively long half-life [44]. In the future, pairing NG2 with apoptotic markers would reduce this concern. For the current work, minimal pericyte change is supported by the other 5 metrics also showing little change and the viability of the capillaries in vivo [18]. Furthermore, Barber et al. did not find significantly more pericyte ghosts in hyperglycemic mice [17]. However, they did find changes in microglia, astrocytes, retinal thickness, number of apoptotic cells and number of acellular capillaries compared to healthy mice by 36 weeks, suggesting DR related pathology. Other studies using the Ins2^Akita^ have also found evidence of changes in pro- and anti-angiogenic factors but not overt vascular changes [45]. Meanwhile, neural cell [46,47] deficits observed in other models may explain deficits in visual acuity and behavioral responses seen in Ins2^Akita^ mice [48]. Combined with our data, this suggests metrics that are not easily accessed in human patients may precede vascular changes.

Our results can also be compared to another popular model, the streptozotocin (STZ) protocol which pharmacologically imparts insulin dysfunction [49,50]. Reports have shown vascular degeneration [46,51,52], visual acuity loss [53], and inflammation [46,53]. However, this model can induce a variable hyperglycemia phenotype [54,55] and has more noted off-target effects such as changes in kidney [56], liver [57], and myocytes [58]. Even within pancreatic tissue it induces widespread cell death through necrosis, DNA damage, release of nitric oxide, and generation of reactive oxygen species [50,59,60]. These changes obscure the relationship between retinal pathology and potential causal factors.

In contrast, we leveraged the consistent hyperglycemia of the Ins2^Akita^ to evaluate BG’s impact on an age range loosely corresponding to young adulthood into “middle-age” when scaled into human years [61]. In humans, poorly managed BG by this age often manifests as strong microvascular remodeling including vascular alterations, pericyte loss [4,62,63], and vessel growth such as IRMAs [64,65] which are highly tortuous. Our data did not show consistent evidence of these vascular abnormalities at any location or at any age. This suggested that elevated BG is not a strong predictor of vascular remodeling within one year. This is especially compelling given these mice do not have commonly present comorbidities seen in humans such as hypertension, hyperlipidemia, or reduced physical activity [12–14]. There are also instances in which humans experience transient worsening of pathology after treatment reduces BG levels [66], suggesting more complex mechanisms than elevated BG alone. In fact, some studies suggest glucose variability may impact pathology more than its elevation [67–69].

The discrepancy between our data and human findings makes it tempting to highlight all of the ways that mice are not the same as humans, including ones as simple as mice are simply shorter-lived than humans that persevere through decades of DM [6,7]. However, from our data we can still conclude elevated BG did not associate with strong microvascular changes. Furthermore, the analysis of 27 mice built confidence that we did not overlook any acute phase of rapid vessel loss compensated for by vessel proliferation. And while one may consider that histology only analyzes individual time points for each mouse, the in vivo AOSLO imaging showed evidence that single locations in single subjects are stable over at least 12 weeks. This is highly attractive for human study as it offers the potential to provide individualized treatment and personalized care based on outcomes within subject. For this work, we were able to clearly track all our metrics except pericyte density in vivo, a marker other labs have started to track at high resolutions [42]. Given that small and subtle changes in vascular patterning may be useful for not only earlier diagnosis [11,30], this methodology is especially promising.

We also recognize this work’s limitations. The first is that we did not include females due to their weak hyperglycemic phenotype in these mice [16] and cannot comment on any sex-related differences. This also begs another question, that estradiol may have a protective effect [70], a line for future investigation. Secondly, this work analyzed small FOVs, and while we did evaluate 124 regions, it is possible that small localized changes were missed by this sampling. We attempted to control for this by systemically sampling the same bands of eccentricity using ROIs equivalent or larger than other work [17,46,71], representing ~0.6% of the retina each. Future work could benefit from more automated counts across detailed flat mount 3D reconstructions, but this may require better data processing or reduced resolution. The current methodology would benefit from larger sample sizes focused on exclusively older mice (>30 weeks) and within vascular beds that showed evidence of susceptibility such as the axial vessels and deeper plexuses. This could then guide longitudinal AOSLO studies to areas that might have minute, but meaningful changes. Another area of future investigation is whether pericyte ghosts and acellular capillaries can be tracked in vivo. The benefit would be strong for unifying seminal work in the field with our study as well as refining a new biomarker to track in vivo. Lastly, we only examined elevated BG meaning there was a slim possibility other retinal pathology like microglia, astrocytes, and inflammation changes were also absent resulting in the limited vascular changes. This seems unlikely given past reports and the severity and duration of the elevated BG. However, this is a clear opportunity for the next study to incorporate those non-vascular metrics and other cofactors such as mitochondrial dysfunction [72] or inflammation [73]. If these changes do precede vascular changes as is suggested by this data, this provides an exciting avenue for intervention prior to any vascular pathology.

In conclusion, elevated blood glucose for a year in Ins2^Akita^ mice did not associate with notable changes in six vascular metrics. This detailed anatomical study mirrored our prior report that vascular perfusion at the single blood cell level is also unchanged in the Ins2^Akita^ [18]. Further work is necessary to reveal any interdependence of hyperglycemia with other cofactors in DR-related microvascular change.

## Supporting information

S1 Fig All euglycemic Z-Stacks and traces. Eccentricity increases from left to right and age from top to bottom. Each row is a different mouse with the age shown in weeks at left. Color-coded traces were overlayed on top. Scale bar = 25 μm.
(TIFF)

S2 Fig All hyperglycemic Z-Stacks and traces. Eccentricity increases from left to right and age from top to bottom. Each row is a different mouse with the age shown in weeks at left. Combined with S1 Fig, this shows the entire analyzed ex vivo data set. Color-coded SNT traces overlayed on top. Scale bar = 25 μm.
(TIFF)

S3 Fig Little eccentricity dependent blood glucose effect on any vascular metrics. Quantification of (A) vessel density, (B) vessel branching, (C) vessel lengths, and (D) tortuosity by vascular layer and eccentricity. Two-way ANOVAs with post-hoc Tukey’s *P < 0.05.
(TIFF)

S4 Fig Little age dependent blood glucose effect on any vascular metrics. Quantification of (A) vessel density, (B) vessel branching, (C) vessel lengths, and (D) tortuosity by vascular layer and age. Two-way ANOVAs with post-hoc Tukey’s *P < 0.05.
(TIFF)

S5 Fig Pericyte density was not affected by eccentricity or age. Quantification of pericyte density across trilaminar layers and (A) eccentricity and (B) age. Two-way ANOVAs with post-hoc Tukey’s *P < 0.05.
(TIFF)

S6 Fig Axial vessel composition was not affected by eccentricity or age. Quantification of axial vessels across trilaminar layers and (A) eccentricity and (B) age. Two-way ANOVAs with post-hoc Tukey’s.
(TIFF)

S7 Fig Vessel density is similar between euglycemic and hyperglycemic mice. (A) Euglycemic and hyperglycemic in vivo max projections of 4 different locations at 24 weeks of age. Contrast adjusted; originals available upon request. ImageJ SNT traces were exported and overlayed on top. Eccentricity increases from top to bottom. Scale bar = 10 μm (B) Blood glucose measurements for the two mice. (C) Vessel density quantification.
(TIFF)

S8 Fig Weak correlations between vascular metrics and age or BG duration. Quantification of vascular metrics vs (A) age and (B) duration of BG. The top 4 graphs plot total, superficial, intermediate, and deep layers while the bottom 4 plot SI and ID regions. Metrics are: vessel density, vessel branching, vessel length, and tortuosity.
(TIFF)

S9 Fig Weak correlations between hyperglycemic vascular metrics and BG level, age, or BG duration. Quantification of vascular metrics vs (A) BG level, (B) age, and (C) BG duration. The top 4 graphs plot total, superficial, intermediate, and deep layers while the bottom 4 plot SI and ID regions. Metrics are: vessel density, vessel branching, vessel length, and tortuosity.
(TIFF)

S1 Appendix T-Tests, ANOVAs, and post-hoc analysis. (XLSX)

S2 Appendix Correlation and linear regression. (XLSX)

S1 Video Euglycemic and hyperglycemic activity. Overhead recording of 22-week-old mice placed within a clean cage showed similar size and activity levels.
(MP4)
