# Supplementary figures and images for: One year of hyperglycemia in the Ins2Akita mouse does not impart changes in retinal vascular patterning
Source: PLoS One. 2026 May 28;21(5):e0348363. doi: 10.1371/journal.pone.0348363 (PMC13218519; doi:10.1371/journal.pone.0348363)

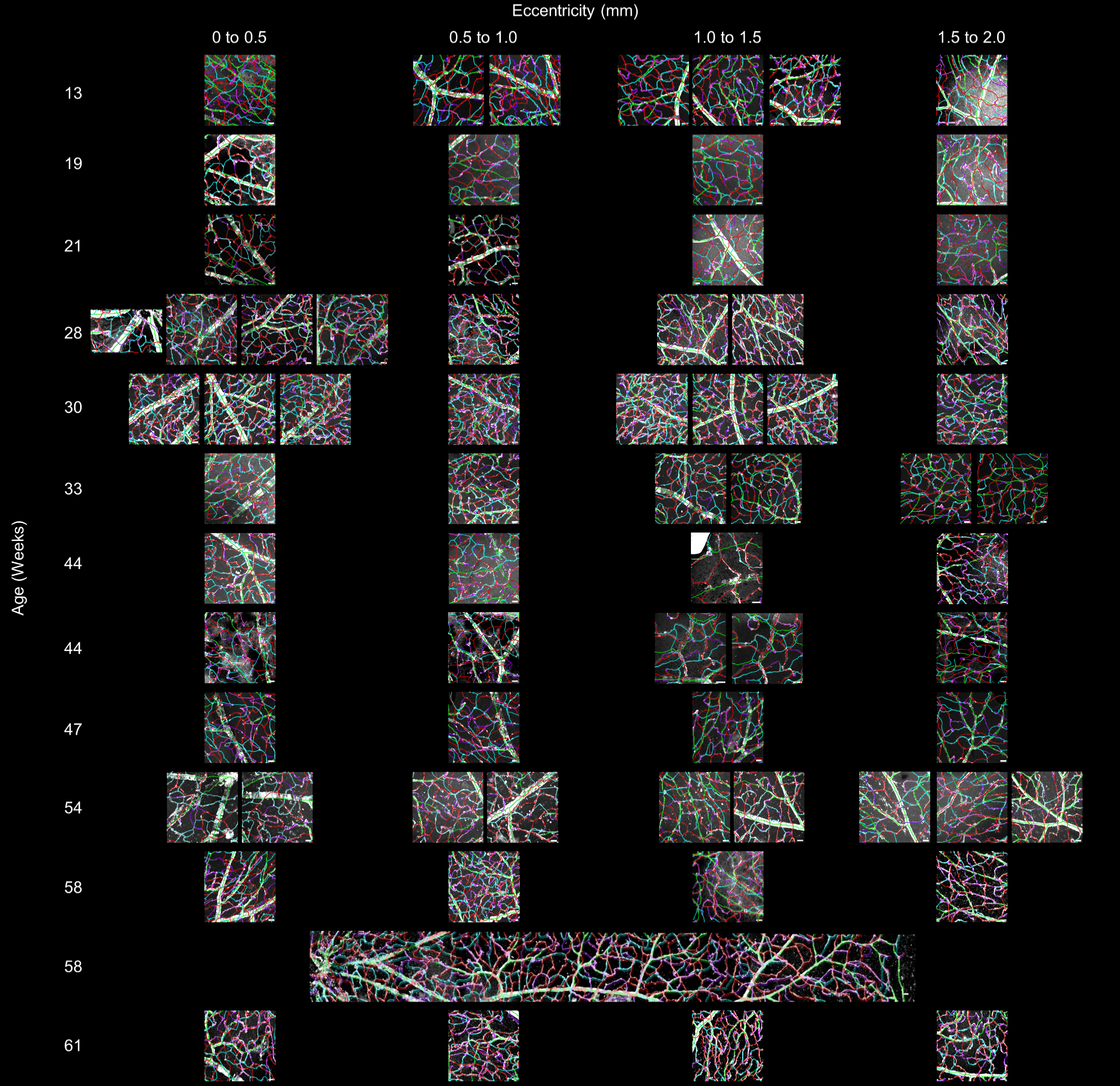

Supplement: S1 Fig — Eccentricity increases from left to right and age from top to bottom. Each row is a different mouse with the age shown in weeks at left. Color-coded traces were overlayed on top. Scale bar = 25 μm. (TIFF) [file pone.0348363.s001.tiff]

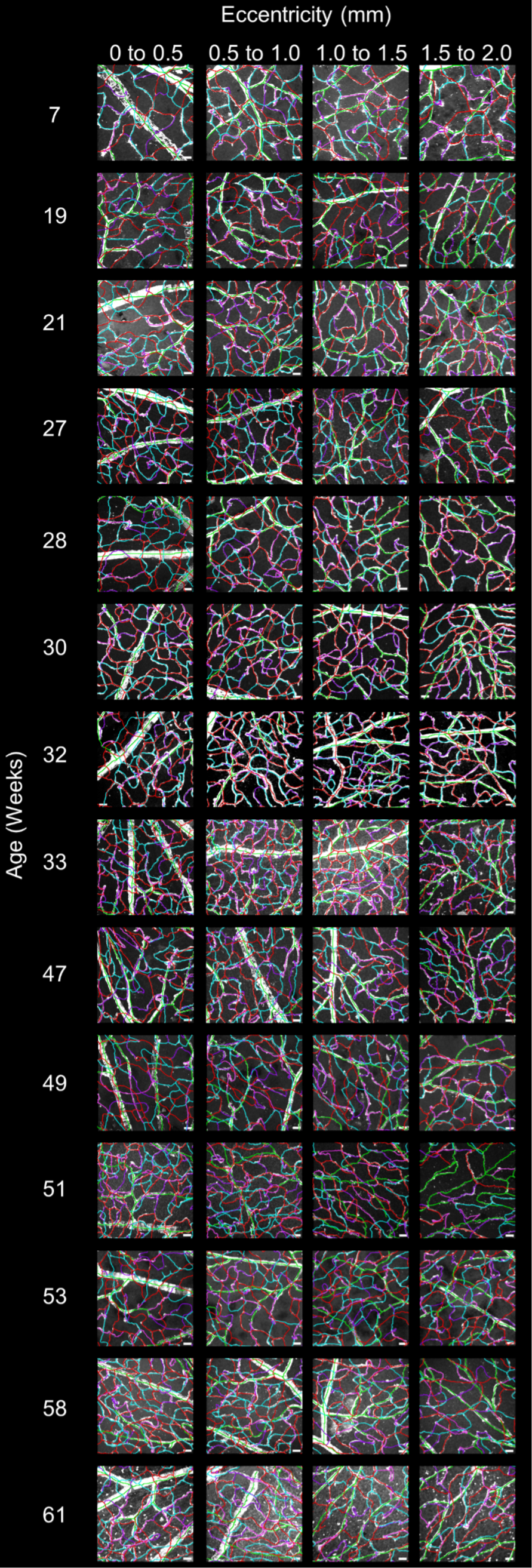

Supplement: S2 Fig — Eccentricity increases from left to right and age from top to bottom. Each row is a different mouse with the age shown in weeks at left. Combined with S1 Fig, this shows the entire analyzed ex vivo data set. Color-coded SNT traces overlayed on top. Scale bar = 25 μm. (TIFF) [file pone.0348363.s002.tiff]

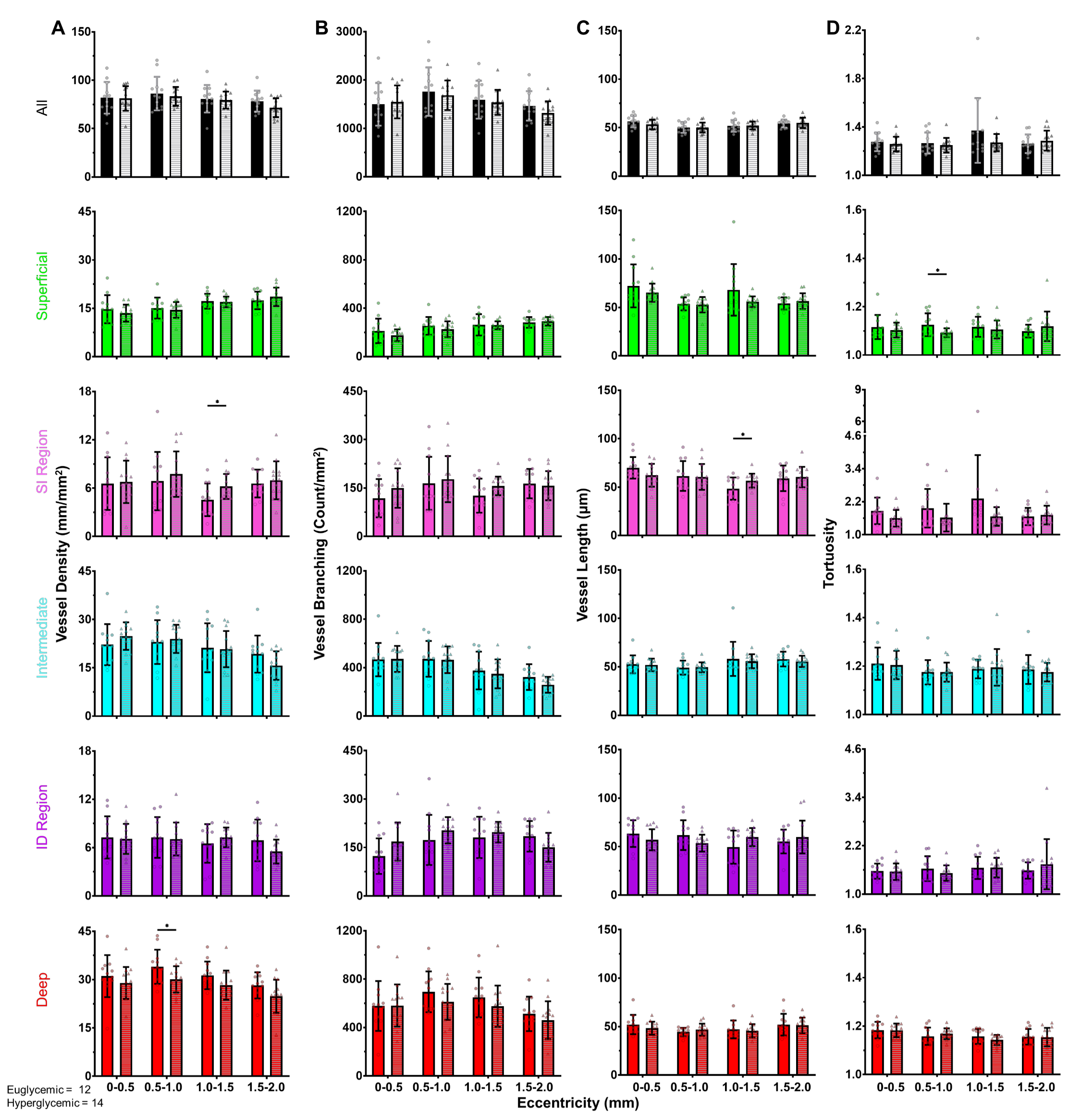

Supplement: S3 Fig — Quantification of (A) vessel density, (B) vessel branching, (C) vessel lengths, and (D) tortuosity by vascular layer and eccentricity. Two-way ANOVAs with post-hoc Tukey’s *P < 0.05. (TIFF) [file pone.0348363.s003.tiff]

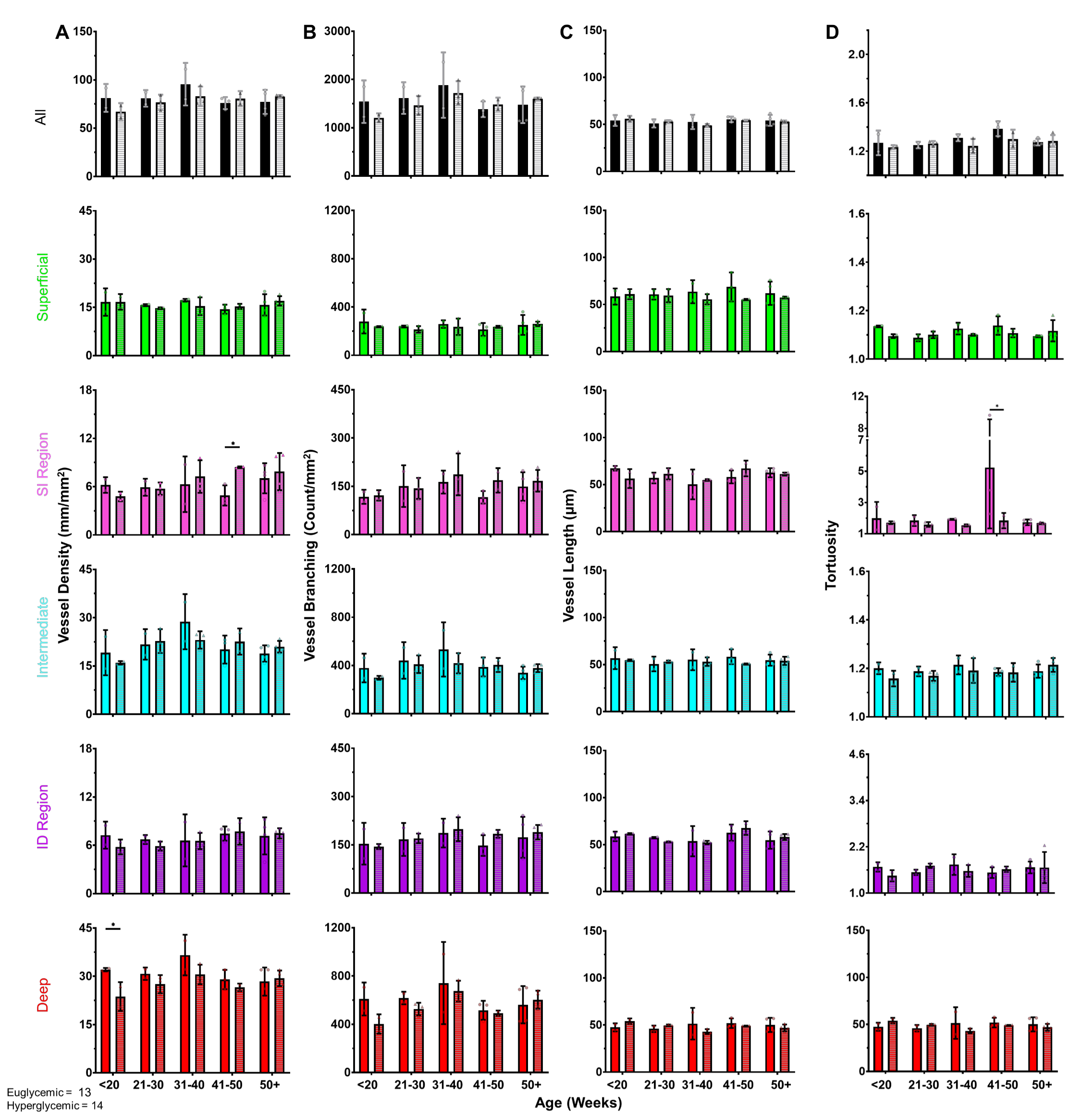

Supplement: S4 Fig — Quantification of (A) vessel density, (B) vessel branching, (C) vessel lengths, and (D) tortuosity by vascular layer and age. Two-way ANOVAs with post-hoc Tukey’s *P < 0.05. (TIFF) [file pone.0348363.s004.tiff]

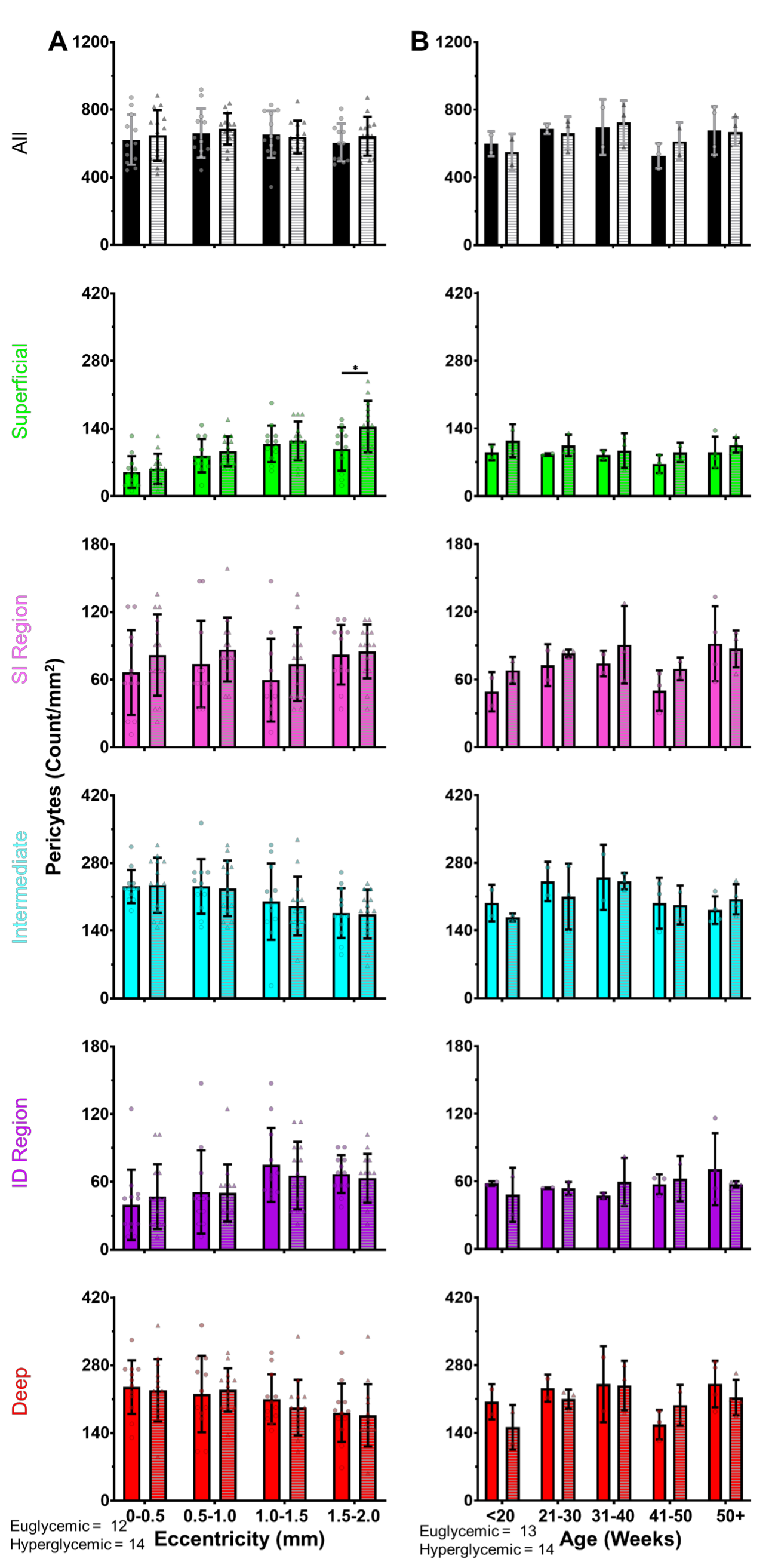

Supplement: S5 Fig — Quantification of pericyte density across trilaminar layers and (A) eccentricity and (B) age. Two-way ANOVAs with post-hoc Tukey’s *P < 0.05. (TIFF) [file pone.0348363.s005.tiff]

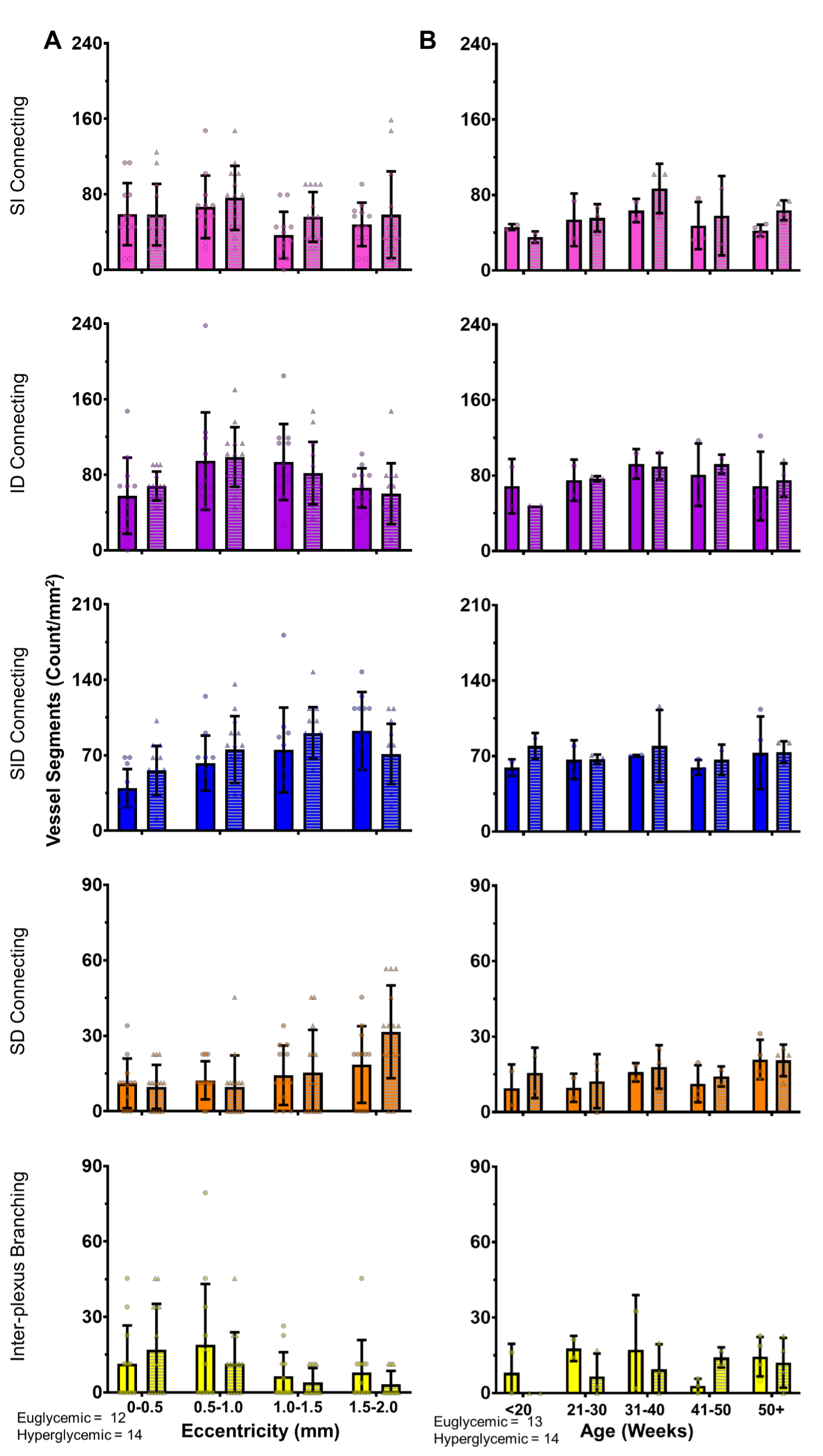

Supplement: S6 Fig — Quantification of axial vessels across trilaminar layers and (A) eccentricity and (B) age. Two-way ANOVAs with post-hoc Tukey’s. (TIFF) [file pone.0348363.s006.tiff]

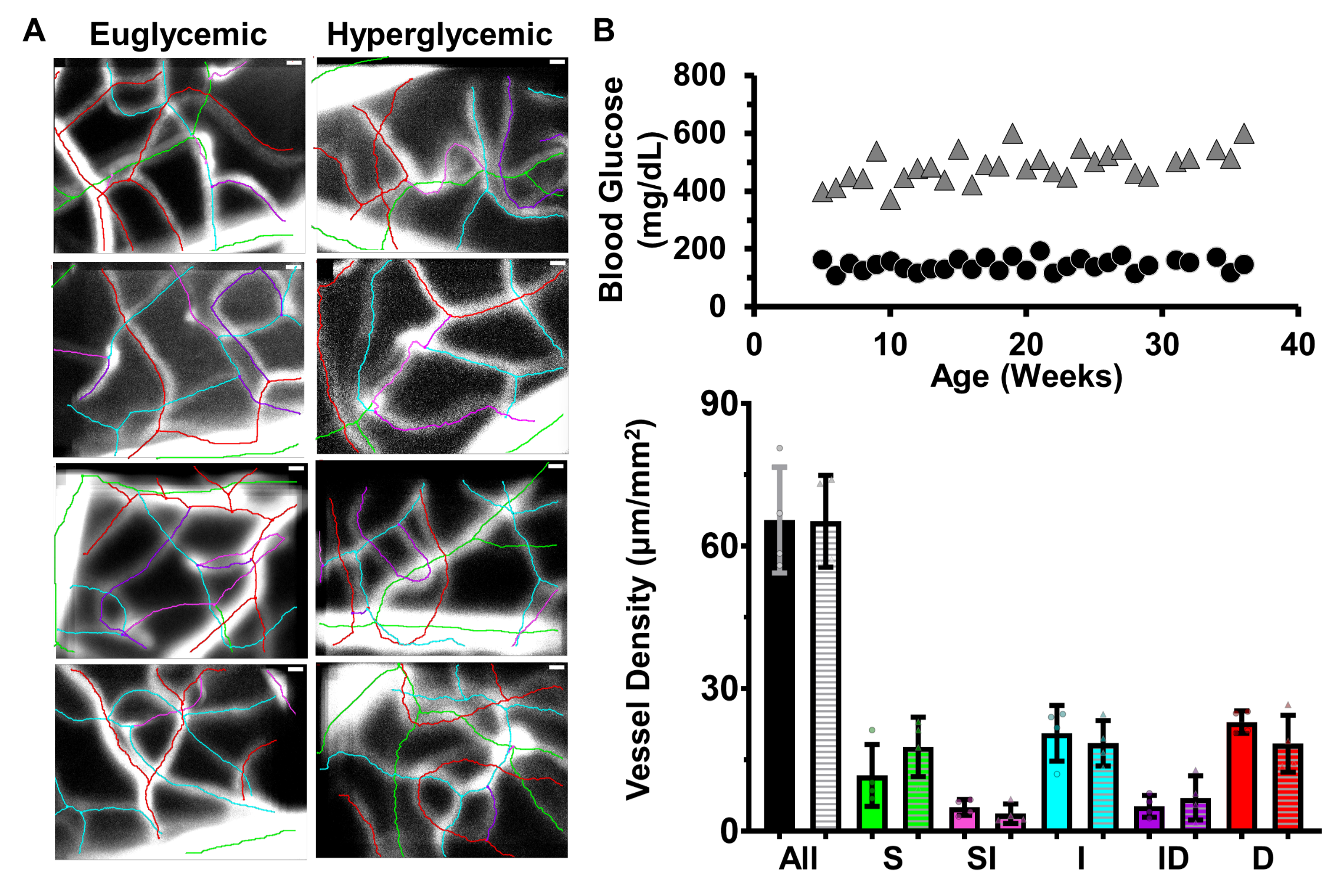

Supplement: S7 Fig — (A) Euglycemic and hyperglycemic in vivo max projections of 4 different locations at 24 weeks of age. Contrast adjusted; originals available upon request. ImageJ SNT traces were exported and overlayed on top. Eccentricity increases from top to bottom. Scale bar = 10 μm (B) Blood glucose measurements for the two mice. (C) Vessel density quantification. (TIFF) [file pone.0348363.s007.tiff]

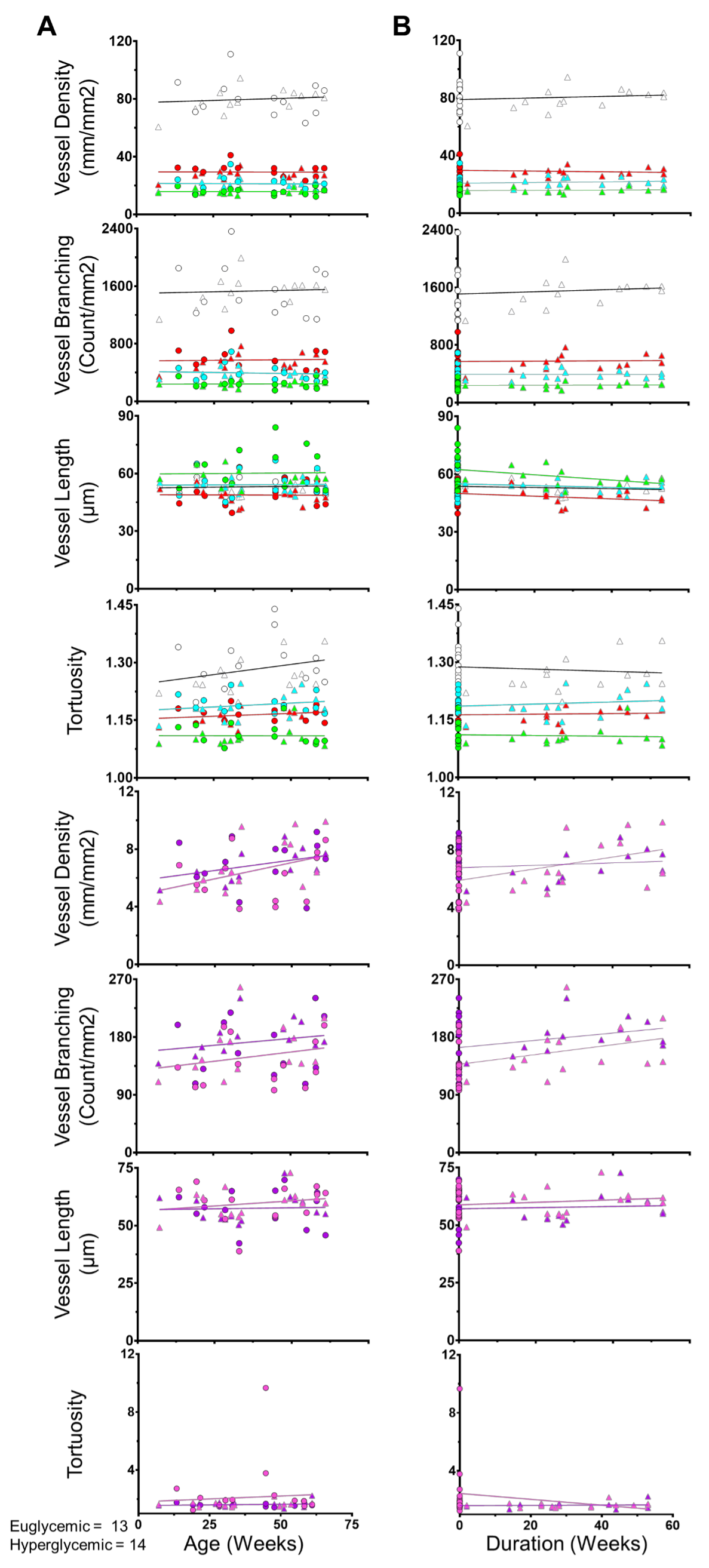

Supplement: S8 Fig — Quantification of vascular metrics vs (A) age and (B) duration of BG. The top 4 graphs plot total, superficial, intermediate, and deep layers while the bottom 4 plot SI and ID regions. Metrics are: vessel density, vessel branching, vessel length, and tortuosity. (TIFF) [file pone.0348363.s008.tiff]

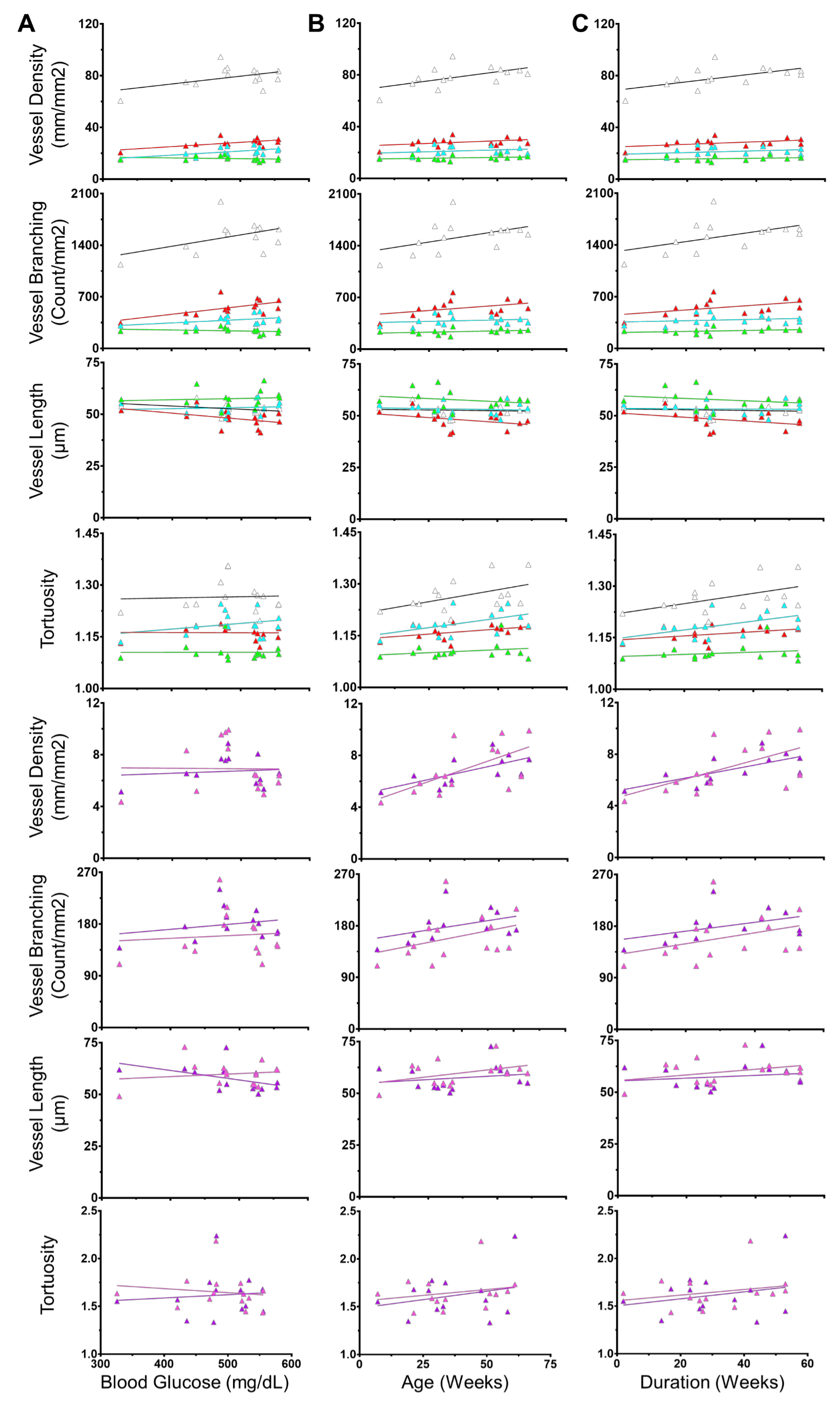

Supplement: S9 Fig — Quantification of vascular metrics vs (A) BG level, (B) age, and (C) BG duration. The top 4 graphs plot total, superficial, intermediate, and deep layers while the bottom 4 plot SI and ID regions. Metrics are: vessel density, vessel branching, vessel length, and tortuosity. (TIFF) [file pone.0348363.s009.tiff]
